# Author’s reply

**Published:** 2011-02

**Authors:** Sangeeta Sharma

**Affiliations:** Department of Neuropsychopharmacology, Institute of Human Behaviour and Allied Sciences, India. E-mail: sharmasangeeta2003@gmail.com

Sir,

This is with reference to the comments on our article[1]. The limitation of the study is mentioned in the discussion. Similar experiences have been reported by other authors in this area[2–3] where a response rate ranging from 38%-70% was reported. We agree with the comments regarding generalization of findings from such studies and suggested measures to increase the response rate. Due care was taken with regard to design and layout of the questionnaire and pilot testing. A well sensitized, trained post graduate researcher collected the data after seeking appointment with the respondents recognizing time constraints etc.

Despite a low response rate, we feel the data is important to be shared as it reflects the initial trends pertaining to a very important but neglected area. If it is able to inspire more such studies the publication would have achieved at least a part of its objective i.e., eliciting the attitude and opinion towards essential medicine formulary to bring about further positive change. A low response rate may also be indication of a primary indifference towards the subject. Response elicited by persuasive methods may not be true and honest. We feel that the data presented reflects a primary uninfluenced response.
